# Mixed-methods evaluation of a rights-based intervention to reduce long-term segregation in mental health services: the HOPE(S) National Programme in England

**DOI:** 10.1136/bmjopen-2025-115706

**Published:** 2026-10-15

**Authors:** Alina Haines-Delmont, Kathryn Fradley, Dineesha Georgeena Rajan, Dawn Cavanagh

**Affiliations:** 1 Nursing and Public Health, Manchester Metropolitan University, Manchester, Greater Manchester, UK; 2 School of Nursing and Public Health, Manchester Metropolitan University, Manchester, Greater Manchester, UK; 3 Department of Social Care and Social Work, Manchester Metropolitan University, Manchester, Greater Manchester, UK

**Keywords:** Disabled Persons, Patient Preference, Adult psychiatry, Child & adolescent psychiatry, Forensic psychiatry, Quality of Life

## Abstract

**Objectives:**

To evaluate the implementation and outcomes of HOPE(S), a national rights-based intervention designed to reduce long-term segregation (LTS) for autistic people, people with a learning disability and children and young people in mental health inpatient services in England.

**Design:**

Pre-post, single-group mixed-methods evaluation combining quantitative analysis of routine clinical and self-report outcomes with qualitative interviews, focus groups and written submissions. Reflexive thematic analysis was undertaken with co-production and lived experience sense-checking.

**Setting:**

National Health Service (NHS)-commissioned inpatient mental health services across England, delivered across multiple secondary and tertiary care providers between May 2022 and March 2025.

**Participants:**

The quantitative cohort comprised 122 individuals in LTS who received HOPE(S) support, 11 family members who engaged in trauma-informed therapy and 388 clinical staff who completed at least part of the Professional Quality of Life; paired staff analyses included 64-68 participants. Qualitative data were collected from 73 stakeholders, including individuals with lived experience, family members, clinical staff, intervention practitioners, commissioners and regulators. Participants were identified through routine service referral and professional networks.

**Intervention:**

HOPE(S) is a multi-component intervention comprising practitioner training in rights-based and trauma-informed care, structured assessment of barriers to change and ongoing therapeutic and practice-leadership support for individuals in LTS and their clinical teams. Family members were offered trauma-informed therapy and access to peer support. Duration of involvement varied according to clinical need and service context.

**Primary and secondary outcome measures:**

Quantitative outcomes included quality of life, use of restrictive practices, access to fresh air, engagement in meaningful activity, staff well-being and family functioning.

Qualitative outcomes focused on experiences of LTS, perceived impacts of HOPE(S) and contextual factors influencing implementation.

**Results:**

Of 122 individuals who received HOPE(S), 83 (68%) moved out of LTS at least once during the evaluation period, with 24 discharged into the community. Among those with paired data, physical restraint decreased from 14.62 to 6.23 incidents/month (n=58, t(57)=2.73, p=0.008), chemical restraint decreased according to the Wilcoxon signed-rank test (n=57, z=2.01, p=0.045; median=0 at both time points) and seclusion decreased from 0.53 to 0.22 incidents/month (n=59, t(58)=3.01, p=0.004). Mechanical restraint decreased from 0.34 to 0.07 incidents/month, but this was not statistically significant (n=59, t(58)=1.17, p=0.247). Access to fresh air increased from 7.69 to 14.09 days/month (n=57, t(56)=4.18, p<0.001) and meaningful activities increased from 3.11 to 21.70 /month (n=56, t(55)=5.79, p<0.001). Quality-of-life scores improved from 25.33 to 17.56 among nine individuals with complete paired Mini Maslow Assessment of Needs Scale for Learning Disabilities (Mini-MANS-LD) data (t(8)=3.32, p=0.011), although this finding should be interpreted cautiously given the small sample. Among staff with paired Professional Quality of Life (ProQOL -5) data, compassion satisfaction increased from 38.13 to 40.31 (n=64, t(63)=4.20, p<0.001), burnout decreased from 24.66 to 22.28 (n=68, t(67)=4.71, p<0.001) and secondary traumatic stress decreased from 22.47 to 19.93 (n=68, t(67)=3.25, p=0.002). Among eight family members with repeated 15-item Systematic Clinical Outcomes and Routine Evaluation data, none met the criteria for reliable or clinically significant improvement in family functioning. Qualitative findings described LTS as a profoundly isolating experience and HOPE(S) as supporting relational safety, shifts in care culture and renewed hope for families and staff, while also identifying persistent systemic and organisational barriers to sustaining change.

**Conclusions:**

This is the first large mixed-methods study exploring at length the phenomenon of LTS, suggesting that a rights-based, relational intervention can lead to reductions in restrictive practices, alongside improvements in some individual and staff outcomes. The findings highlight the importance of system-level approaches that prioritise relational safety, dignity and least-restrictive practice. Further research is required to examine the sustainability of change, longer-term outcomes and transferability across settings.

**Trial registration:**

Not applicable.

**STRENGTHS AND LIMITATIONS OF THIS STUDY**

- This is the first study to explore long-term segregation in depth, using a mixed-methods approach that integrates routine quantitative outcomes and validated self-report measures with qualitative data from multiple stakeholder groups.
- The study was embedded within real-world service delivery across diverse mental health inpatient settings, enhancing ecological validity but resulting in variability in data completeness and follow-up timing.
- Meaningful patient and public involvement, including lived experience co-production and sense-checking, strengthened the relevance and credibility of the study design and interpretation.
- The pre-post, single-group design without a comparator limits causal inference and missing pre-post pairs reduced sample sizes for some quantitative analyses; some findings, particularly Mini-MANS-LD quality-of-life outcomes, should therefore be interpreted cautiously.
- The programme was implemented nationally, but the evaluation sample should not be interpreted as nationally representative; ethical and practical constraints precluded experimental designs or recruitment of individuals currently detained in inpatient settings.

## Background

 Restrictive practices such as physical restraint, chemical restraint and seclusion are widely recognised as harmful and are the focus of substantial international efforts to reduce their use in mental health services. Although intended to prevent immediate harm, these practices are not always applied as a last resort, particularly in contexts characterised by trauma-informed cultures, fearful or unsupported staff or environments poorly suited to individuals’ needs.^1–5^ In contrast to the substantial literature on seclusion and restraint, long-term segregation (LTS) has received comparatively little empirical scrutiny, although a growing body of qualitative and regulatory evidence has documented its psychological, relational and human-rights impacts.^6 7^

The Mental Health Act Code of Practice defines LTS as a situation in which a patient is prevented from freely mixing with others on a long-term basis because of a sustained and high risk of harm to others, following a multidisciplinary review and agreement with the responsible commissioning authority.^8^ Statutory guidance requires daily nursing review, weekly multidisciplinary review and monthly external scrutiny by hospital managers or commissioners. Although providers must demonstrate that segregation remains necessary and proportionate, no maximum duration is specified in law, and national reviews show wide variation in how LTS criteria are interpreted and applied in practice.^9 10^ In practice, the physical arrangements associated with LTS vary across services. Individuals may be cared for separately from other patients within a ward or unit, sometimes in a dedicated room or area, with little or no contact with peers and limited contact with staff, family members and the wider community. Some environments provide very limited stimulation or access to ordinary activities, and segregation may continue for extended periods, sometimes months or years. LTS is distinct from seclusion: while seclusion is time-limited and used during acute crises, LTS is a longer-term ‘care’ status focused on sustained risk management and containment. As such, LTS may be unfamiliar to clinicians outside the UK, as comparable jurisdictions often use different terminology or place tighter legal restrictions on similar practices.

The Care Quality Commission has repeatedly raised concerns that LTS is used in environments lacking appropriate therapeutic support, and that its persistence may reflect systemic and organisational failures rather than clinical need.^11 12^ More recent national oversight has continued to identify delayed discharge, weak accountability and difficulties translating independent review recommendations into sustained changes in people’s care and lives.^13^

LTS disproportionately affects autistic individuals, people with a learning disability and children and young people accessing mental healthcare. Individuals in LTS have little or no interaction with peers and often limited contact with family members^8 10^ and emerging qualitative evidence has described profound psychological and relational harm, erosion of personhood, loss of hope and concerns regarding human-rights violations.^6^ They often experience significant psychological, social and physical harm.^10 14^ These harms include institutional trauma, social isolation and the denial of basic human rights, with LTS often enacted in environments ill-suited to recovery, leading to chronic relational disconnection as a coping strategy.^15^ These harms highlight broader systemic issues relating to governance, commissioning and accountability in inpatient services. While international frameworks, including the WHO^16^ and the UN Convention on the Rights of Persons with Disabilities (CRPD)^17^ call for the elimination of coercive practices, there remains limited empirical evidence on scalable, rights-based alternatives to LTS. Few evaluated models exist that offer a whole-systems, relational approach to reducing segregation while improving quality of life, safety and therapeutic culture.

The National HOPE(S) Programme was implemented in 2022 to address these systemic challenges. Rooted in trauma-informed and human-rights principles, HOPE(S) aims to reduce the use of LTS by addressing the cultural, organisational and relational factors that contribute to its persistence. This mixed methods evaluation addresses a significant evidence gap by examining the implementation and outcomes of a nationally implemented rights-based intervention - HOPE(S). Drawing on the Capabilities Approach,^18 19^ the study shifts focus from risk containment to expanding individuals’ freedoms and opportunities to live lives they have reason to value. By situating HOPE(S) within existing service structures, the evaluation provides an applied model of system-level transformation with relevance for international mental health reform.

## Methods

### Aim

The overall aim of the study was to explore experiences and accounts of key stakeholders involved in HOPE(S), as well as to measure changes with regard to key outcomes for people in LTS, families, professionals and services following the introduction of HOPE(S). Given the exploratory, real-world implementation focus of the evaluation and the absence of prior national data on LTS reduction interventions, no formal hypotheses were pre-specified.

### Design and participants

#### Design

This pre-post, single group mixed-methods study covered the implementation of the national HOPE(S) programme - hereby referred to as the ‘intervention’ or ‘HOPE(S)’ - from May 2022 to March 2025 in National Health Service (NHS) England-commissioned mental health services.

#### Qualitative participants and data collection

Qualitative data were collected between July 2023 and January 2025 in two waves using semi-structured interviews, focus groups and, where required for accessibility, written responses. In total, 73 unique participants contributed qualitative data across 50 interviews, 9 focus groups and 2 written responses. Participants included families of individuals in LTS, individuals with lived experience of LTS and/or other restrictive practices, clinical staff, intervention staff (HOPES and Respond practitioners), service/regional managers, commissioners and regulators. Some participants contributed to more than one session but were counted only once in the overall sample. For more information, see online supplemental material/SM (SM 4.3).

A detailed qualitative analysis of the impact and human-rights implications of LTS using this qualitative dataset has subsequently been published separately^6^; the present study reports these data within the wider mixed-methods evaluation of HOPE(S).

#### Quantitative data sources

Secondary analysis of routine clinical data and self-report outcome measures collected by intervention staff between 3 May 2022 and 17 March 2025 for up to 122 individuals who received HOPE(S); 11 family members who engaged with trauma informed therapy (Respond) and clinical staff involved in HOPE(S). In total, 388 staff completed at least part of the ProQOL-5. Complete baseline subscale data were available for 376 staff for compassion satisfaction, 379 for burnout and 377 for secondary traumatic stress. Complete matched baseline and follow-up data were available for 64 staff for compassion satisfaction and 68 for burnout and secondary traumatic stress. The ProQOL-5 was made available to staff participating in HOPE(S) training or practice-support activity rather than being distributed to a fixed, enumerated cohort; completion was voluntary. Consequently, the total number of staff who could potentially have completed the measure was not recorded.

Inclusion criteria for quantitative analyses were receipt of HOPE(S) support and availability of both a baseline and follow-up data; no additional exclusion criteria were applied. For clarification, n=122 represents the total number of individuals receiving HOPE(S) support, whether this was intensive support or consultancy only support. Demographic data and a limited number of outcome measures (not reported here, eg, physical health measures) were collected for all individuals, regardless of level of support (n=122). But for measures relating to quality of life, access to fresh air, engagement in meaningful activities, mechanical restraint, physical restraint, chemical restraint, seclusion and physical health checks, the data were collected only for individuals receiving intensive support (n=60). Some minimal cross-over (a few cases) from consultancy to intensive and vice-versa might have happened over the course of the HOPE(S) intervention. This means that the level of support at the time data were collected may not have remained consistent throughout the individual’s experience. As a result, data may have been collected when an individual was receiving intensive support but not at other points when they were receiving consultancy support, or vice versa.

### Demographics of HOPE(S) participants

The mean age of individuals receiving both types of HOPE(S) support was 27.73 (SD=10.25, range 12–61). Most were male (including trans male) (64.75%, n=79), 33.06% (n=40) female (including trans female), and 1.64% (n=2) non-binary. The majority were white (81.15%, n=99). Some had been detained since childhood and were only later identified as needing support.

### Relationship between qualitative and quantitative samples

The qualitative and quantitative components were not designed as formally linked cohorts. Some overlap may have occurred, particularly among clinical staff who completed the ProQOL and also participated in a qualitative interview or focus group; however, participant identifiers were not collected in a way that permitted linkage across the two datasets in order to protect anonymity. Consequently, the extent of any overlap between the qualitative and quantitative samples cannot be determined.

### Other

Sense checking and co-production sessions with people with lived experience, families and other stakeholders were undertaken to validate findings and identify recommendations for future policy, practice and research. For more detail on data collection, see online supplemental material. Full findings are presented in the final report.^20^

### Intervention

The programme was built on a clinical model of care known as the HOPE(S) model,^21^ based on a philosophy of person-centred, human rights-based positive care. It enables clinical teams and individuals in LTS to understand the significant barriers and systemic issues maintaining their circumstances. It includes three key components: (1) HOPE(S) training, (2) the Barriers to Change Checklist and (3) intervention by HOPE(S) practitioners for individuals in LTS and their staff teams. For detailed information about the model, see *online supplemental material SM (SM1-2)*. The programme also includes family-focused interventions, such as trauma-informed therapy delivered to family members by ‘Respond’ (a national charity: https://respond.org.uk/) and a HOPE(S) family peer support forum for those with children, siblings or friends in LTS.

### Setting

HOPE(S) was rolled out in May 2022 to 40 NHS-commissioned organisations, comprising 68 mental health hospitals providing inpatient psychiatric care for children and young people, adults with a learning disability or autistic individuals across all regions of England. These settings included specialist inpatient services, including secure services, providing assessment, treatment and ongoing psychiatric care, rather than acute medical inpatient care. The national programme ended in March 2025. For a summary of the HOPE(S) implementation logic model and outputs, see figure 1.

**Figure 1:**
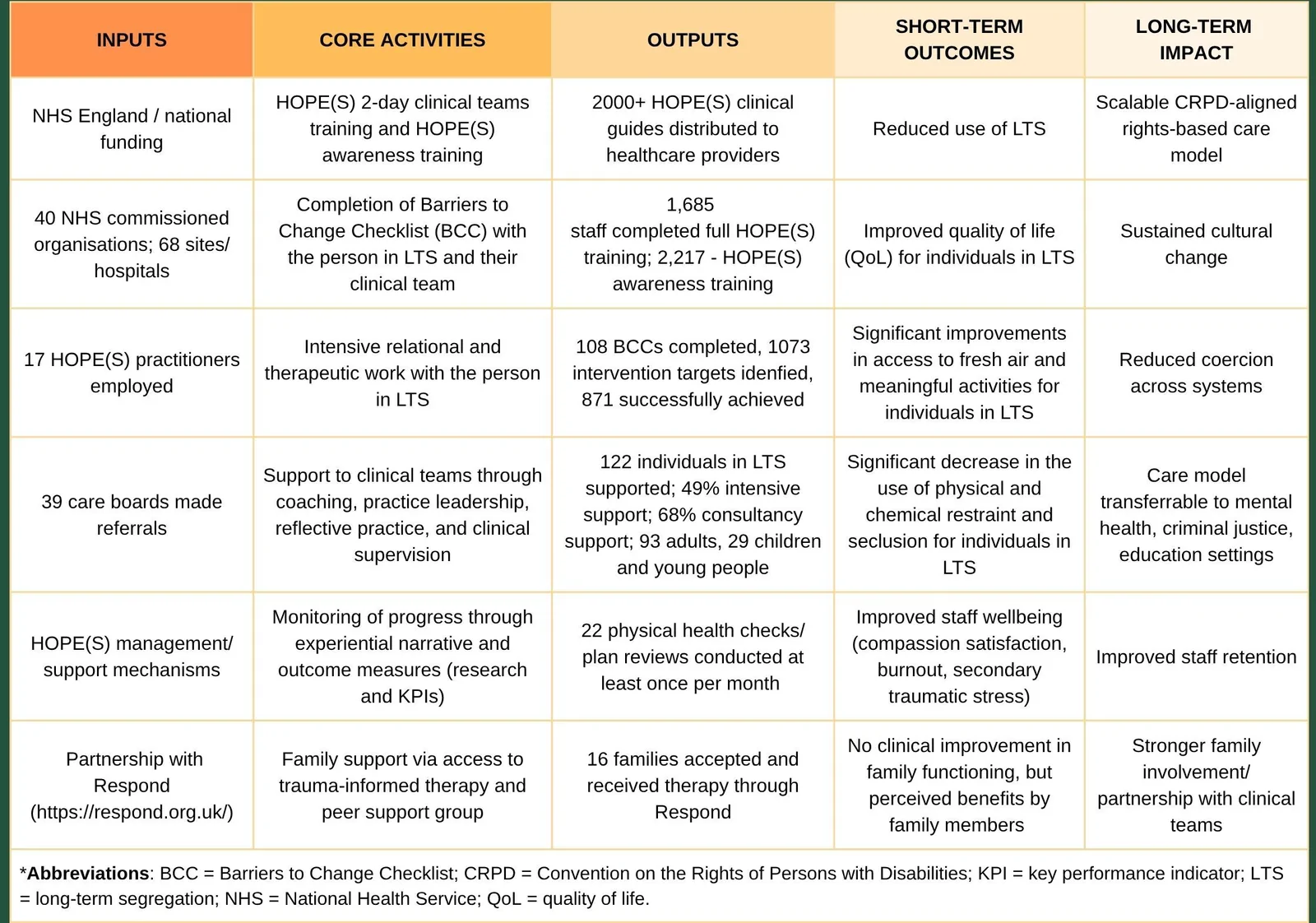
Implementation of HOPE(S). Overview of the implementation of the HOPE(S) programme, including its core components, processes and intended outputs. Abbreviations: BCC=Barriers to Change Checklist; CRPD=Convention on the Rights of Persons with Disabilities; KPI=key performance indicator; LTS=long term segregation; NHS=National Health Service; QoL=quality of life.

## Measures and analyses

### Quantitative outcomes

#### Quality of life of individuals in LTS

was measured by Mini-MANS-LD.^22^ The Mini-MANS-LD is a validated, 9-item accessible self-report tool adapted from the original 19-item MANS-LD.^23^ The tool features items such as ‘Are you happy with how you spend your time?’ and ‘Do you try to hurt yourself?’, evaluated on a 5-point Likert scale supported by pictorial and verbal prompts. Higher Mini-MANS-LD scores indicate poorer quality of life. Psychometric evaluations^22^ demonstrate its reliability and validity for adults with intellectual disabilities, reporting a fair internal consistency (alpha=0.74) and good construct validity via significant convergence with the personal well-being index-intellectual disability (PWI-LD; (r=0.67, p<0.001). Clinical teams selected the quality-of-life measure considered most appropriate for each individual. Several measures were therefore used within the programme; Mini-MANS-LD is reported here because it provided the largest sample with analysable paired data. Its validation evidence relates primarily to adults with learning disabilities, and caution is therefore required when considering its applicability to the broader HOPE(S) population. The tool was completed by the individuals in LTS supported by clinical staff. A total score was created by summing individual item scores.

#### Quality of life of clinical staff

was measured using the 30-item ProQOL 5 (20),^24^ assessing compassion satisfaction, burnout and secondary traumatic stress. The ProQOL-5 is a widely used measure of ProQOL comprising compassion satisfaction, burnout and secondary traumatic stress subscales. Published studies support aspects of its reliability, particularly the compassion satisfaction subscale, although concerns have been raised regarding its factor structure and the distinction between burnout and secondary traumatic stress.^25^ Internal consistency was not estimated for the present analytical sample. Staff engaged in HOPE(S) completed the scale. Sub-scale scores were calculated by summing the relevant items.

#### Family functioning

was assessed with the 15-item Systematic Clinical Outcomes and Routine Evaluation (SCORE-15;^26^). Published validation studies report good internal consistency (α=0.90), test-retest reliability and construct validity.^27^ Participants who received therapy from ‘Respond’ (https://respond.org.uk/) completed the tool. Scores were reversed where appropriate and summed. Change was assessed using the clinical/reliable change criteria, rather than a paired t-test.

#### LTS status

was monitored through routine data collection when individuals moved out of LTS, including whether they were discharged into the community. Follow-up data at 3- and 6-month post-intervention recorded whether individuals remained out of LTS.

#### Use of restrictive practices

(physical, chemical, mechanical restraint and seclusion) was documented using clinical records. The monthly average number of restrictive practices was calculated.

#### Access to fresh air

was measured by the number of days per month individuals accessed outdoor or community spaces.

#### Engagement in meaningful activities

was co-defined with individuals in LTS. Activities included physical activity (e.g., gym), therapeutic sessions, family contact, personal care (e.g., haircut, bath), community leave, independence skills (e.g., going shopping or cooking), education and other personally valued experiences (e.g., access to phone calls). The number of meaningful activities per month was recorded.

### Analyses

Quantitative data were analysed using descriptive and inferential statistics (paired t-tests) in SPSS.^28^ Descriptive statistics (mean, SD, range) were calculated for quality of life, access to fresh air, engagement in meaningful activities and use of restrictive practices. Paired t-tests were conducted where sample sizes allowed. Analyses were conducted on complete pre-post pairs only; patterns of missing data are described in the online supplemental material SM (SM3). Normality was assessed using the Shapiro-Wilk test. Normality of paired differences was assessed using the Shapiro-Wilk test. Where normality assumptions were not met for restrictive-practice outcomes, Wilcoxon signed-rank tests were additionally conducted as sensitivity analyses. Findings were consistent with the paired t-tests except for chemical restraint; the Wilcoxon result is therefore reported for that outcome. All paired t-test p values reported in the manuscript are two-sided. Staff follow-up completion was substantially lower than baseline, resulting in high loss to follow-up for the ProQOL-5 analyses. The total number of staff eligible to participate was unknown.

Qualitative data were analysed using reflexive thematic analysis,^29 30^ with co-production and sense-checking integrated into theme development and interpretation. Recruitment continued until no new conceptual insights were identified across stakeholder groups.

### Patient and public involvement

People with lived experience of mental health inpatient care, LTS and family members were involved from the earliest stages of the research. A lived experience advisory panel contributed to the development of the study aims, research questions and protocol prior to data collection, ensuring the evaluation focused on outcomes and experiences considered meaningful by those most affected. Research questions and outcome measures were informed by lived experience priorities, particularly around quality of life, relational safety, dignity, access to fresh air and meaningful activity and the impact of restrictive practices on families and staff. Several standardised outcome measures were reviewed with people with lived experience; some were adapted or excluded where they were considered burdensome, inaccessible or insufficiently reflective of lived priorities.

People with lived experience were actively involved in the design of the study, including decisions about recruitment and data collection. It was agreed that individuals currently receiving the HOPE(S) intervention or detained in inpatient settings should not be approached for research participation, to minimise burden and avoid conflating care with research. As a result, recruitment of people with lived experience focused on individuals who were living in the community and were engaged through trusted charities, advocacy networks and peer connections. Lived experience contributors also informed the conduct of the study, including the co-development of accessible participant information materials, interview guides, consent processes and approaches to safeguarding. Peer researchers and lived experience co-authors were involved in sense-checking emerging findings and interpretations, helping to ensure credibility and relevance. Participants and lived experience contributors were consulted on the perceived burden of participation, including time commitments and emotional impact and adjustments were made accordingly (e.g., flexible interview formats, optional written submissions and the use of remote methods).

People with lived experience and family members have been and continue to be involved in dissemination activities, including co-developing Plain English summaries, contributing to conference presentations and sharing findings through advocacy organisations, professional networks and lived experience forums. Decisions about what findings to prioritise, how they are communicated and to whom, are being made collaboratively to ensure accessibility and impact beyond academic audiences.

### Findings

Quantitative and qualitative data were drawn from multiple sources collected during the national implementation of HOPE(S) between May 2022 and March 2025.

Routine clinical and self-report quantitative data were available for up to 122 individuals who received HOPE(S) support, 11 family members who engaged in trauma-informed therapy (Respond) and 388 clinical staff who completed at least part of the ProQOL-5. Staff paired analyses included 64 participants for compassion satisfaction and 68 for burnout and secondary traumatic stress. Pre-post data availability varied by outcome measure (as detailed in table 1 and online supplemental material SM3), reflecting the real-world nature of implementation and differences in data collection time points; analyses were therefore conducted on complete pre-post pairs for each outcome. Pre- and post-intervention measurement periods also varied by outcome due to differences in routine data availability and timing of intervention engagement.

**Table 1:** Quantitative outcomes: types of restrictive practice, quality of life, access to fresh air and engagement in meaningful activities pre and post HOPE(S) intervention*

Measure	N/paired n	Pre	Post	Mean difference* (df)	Test statistic	Two-sided P	D	95% CI
Restrictive practices** (incidents/month)								
Mechanical restraint	60/59	0.34 (1.87)	0.07 (0.35)	0.27 df=58	t=1.17	0.247	0.15	−0.19 to 0.73
Physical restraint	60/58	14.62 (28.69)	6.23 (13.57)	8.39 df=57	t=2.73	**0.008**	0.36	2.24 to 14.54
Chemical restraint	60/57	Median=0.00	Median=0.00	–	z=2.01	**0.045**	–	–
Seclusion	60/59	0.53 (1.10)	0.22 (0.62)	0.32 df=58	t=3.01	**0.004**	0.39	0.11 to 0.53
Quality of life (Mini-MANS-LD score)***	60/9	25.33 (6.36)	17.56 (5.25)	7.78 df=8	t=3.32	**0.011**	**1.11**	2.37 to 13.18
Access to fresh air (days/month)	60/57	7.69 (12.01)	14.09 (12.43)	−6.40 df=56	t=4.18	**<0.001**	0.55	−9.47 to −3.33
Meaningful activities (number/month)	60/56	3.11 (6.15)	21.70 (23.63)	−18.59 df=55	t=5.79	**<0.001**	0.77	−25.03 to −12.16
Staff quality of life (ProQOL-5 score)†**								
Compassion satisfaction	Unknown***/64	38.13 (6.99)	40.31 (5.48)	−2.19 df=63	t=4.20	**<0.001**	0.53	−3.23 to −1.15
Burnout	Unknown***/68	24.66 (5.45)	22.28 (5.02)	2.38 df=67	t=4.71	**<0.001**	0.57	1.37 to 3.39
Secondary traumatic stress	Unknown***/68	22.47 (5.95)	19.93 (6.17)	2.54 df=67	t=3.25	**0.002**	0.39	0.98 to 4.11

Note 1: SCORE-15 family-functioning outcomes are not included in table 1 because change was evaluated using the clinical and reliable change criteria recommended by Miller *et al*, rather than a paired inferential test. Eight family members had repeated SCORE-15 data; none met the criteria for clinically significant or reliable improvement.

Note 2: Most individual-level outcomes in table 1 were intended to be collected for individuals receiving intensive HOPE(S) support (n=60). In a small number of cases, outcome data were also recorded for individuals receiving consultancy support, and level of support could change during programme involvement. Further details of data availability and completeness are provided in the online supplemental material.

* Mean differences were calculated as pre-HOPE(S) minus post-HOPE(S). For outcomes in which higher values indicate greater frequency or poorer outcomes, a positive mean difference therefore represents a reduction following HOPE(S). For outcomes in which higher values indicate improvement, a negative mean difference represents an increase following HOPE(S).

† Normality assumptions for the paired differences were not met for the restrictive-practice outcomes, and Wilcoxon signed-rank tests were therefore additionally conducted. Findings were consistent with the paired t-tests except for chemical restraint, for which the paired t-test was not statistically significant whereas the Wilcoxon signed-rank test indicated a significant reduction (z=2.01, p=0.045). The chemical-restraint row therefore reports median values and the Wilcoxon result.

‡ Pre- and post-HOPE(S) observation periods differed by outcome. For restrictive practices, pre-HOPE(S) data covered a mean of 7 months (range 0-15) before HOPE(S), and post-HOPE(S) data covered a mean of 10 months (range 1-28) following commencement. For quality of life, baseline Mini-MANS-LD assessment occurred within 4 weeks of HOPE(S) commencing and follow-up at 2-4 months. For access to fresh air and meaningful activities, pre-HOPE(S) data covered a mean of 2 months (range 1-7) before HOPE(S), and post-HOPE(S) data covered a mean of 10 months (range<1-30) following commencement. For staff ProQOL-5 outcomes, baseline assessment occurred within 4 weeks of HOPE(S) commencing and follow-up after a mean of 5 months (range 4-14).

§ Higher Mini-MANS-LD scores indicate poorer quality of life. The analysis was based on only nine complete pre-post pairs and should therefore be interpreted cautiously because of the small sample size and consequent uncertainty in the effect estimate.

¶ In total, 388 staff completed at least part of the ProQOL-5. Complete baseline subscale data were available for 376 staff for compassion satisfaction, 379 for burnout and 377 for secondary traumatic stress. Inferential analyses were restricted to complete matched pre–post data (n=64 for compassion satisfaction; n=68 for burnout and secondary traumatic stress). The total number of staff invited or eligible to complete the ProQOL-5 was not available in the evaluation dataset.

CI, Confidence interval; Mini-MANS-LD, Mini-Maslow Assessment of Needs Scale for Learning Disabilities; ProQOL, Professional Quality of Life.

Qualitative data were collected through 50 semi-structured interviews, 9 focus groups and 2 written responses from 73 unique participants, including individuals with lived experience of LTS, family members, clinical staff, HOPE(S) and Respond practitioners, commissioners and regulators.

### Quantitative outcomes

Of the 122 individuals who received HOPE(S), 83 (68%) moved out of LTS at least once, with 24 discharged directly into the community. Recorded reasons for remaining in LTS during this period included ongoing intervention activity, delayed discharge processes, provider or commissioning constraints and the end of national programme funding. As summarised in table 1, restrictive-practice outcomes generally decreased following HOPE(S). Among individuals with paired data, physical restraint decreased from a mean of 14.62 to 6.23 incidents per month, corresponding to an average reduction of 8.39 incidents per month (t(57)=2.73, p=0.008). Seclusion decreased from 0.53 to 0.22 incidents per month, an average reduction of 0.32 incidents per month (t(58)=3.01, p=0.004). Mechanical restraint also decreased numerically, from 0.34 to 0.07 incidents per month, although this difference was not statistically significant (t(58)=1.17, p=0.247). For chemical restraint, the Wilcoxon signed-rank test indicated a statistically significant reduction (z=2.01, p=0.045), although the median was zero at both time points.

Quality of life, measured using the Mini-MANS-LD, improved among the nine individuals with complete paired data, with mean scores decreasing from 25.33 to 17.56 (mean improvement 7.78 points; t(8)=3.32, p=0.011); however, this finding should be interpreted cautiously given the very small sample. Access to fresh air increased from a mean of 7.69 to 14.09 days per month, an average increase of 6.40 days (t(56)=4.18, p<0.001), while engagement in meaningful activities increased from 3.11 to 21.70 activities per month, an average increase of 18.59 activities (t(55)=5.79, p<0.001).

Among staff with complete paired ProQOL-5 data, compassion satisfaction increased by an average of 2.19 points (t(63)=4.20, p<0.001), while burnout decreased by 2.38 points (t(67)=4.71, p<0.001) and secondary traumatic stress decreased by 2.54 points (t(67)=3.25, p=0.002). Loss to follow-up for staff-reported outcomes was high, and the total number of staff eligible to complete the ProQOL-5 was unknown. For the eight family members with repeated SCORE-15 data following Respond therapy, none met the specified criteria for clinically significant or reliable improvement in family functioning.

### Qualitative findings

Qualitative analysis drew on 49 semi-structured interviews, 8 focus groups and 2 written submissions with 73 stakeholders, including individuals with lived experience of LTS, family members, clinical staff, HOPE(S) practitioners, Respond practitioners, commissioners and regulators. Reflexive thematic analysis identified five interrelated core themes (figure 2) that describe experiences of LTS, perceptions of the HOPE(S) intervention and contextual factors influencing implementation. A more detailed qualitative analysis of the impact and human-rights implications of LTS from this dataset has been reported separately^6^; the themes presented below (with illustrative quotations) focus on the wider mixed-methods evaluation of HOPE(S).

**Figure 2:**
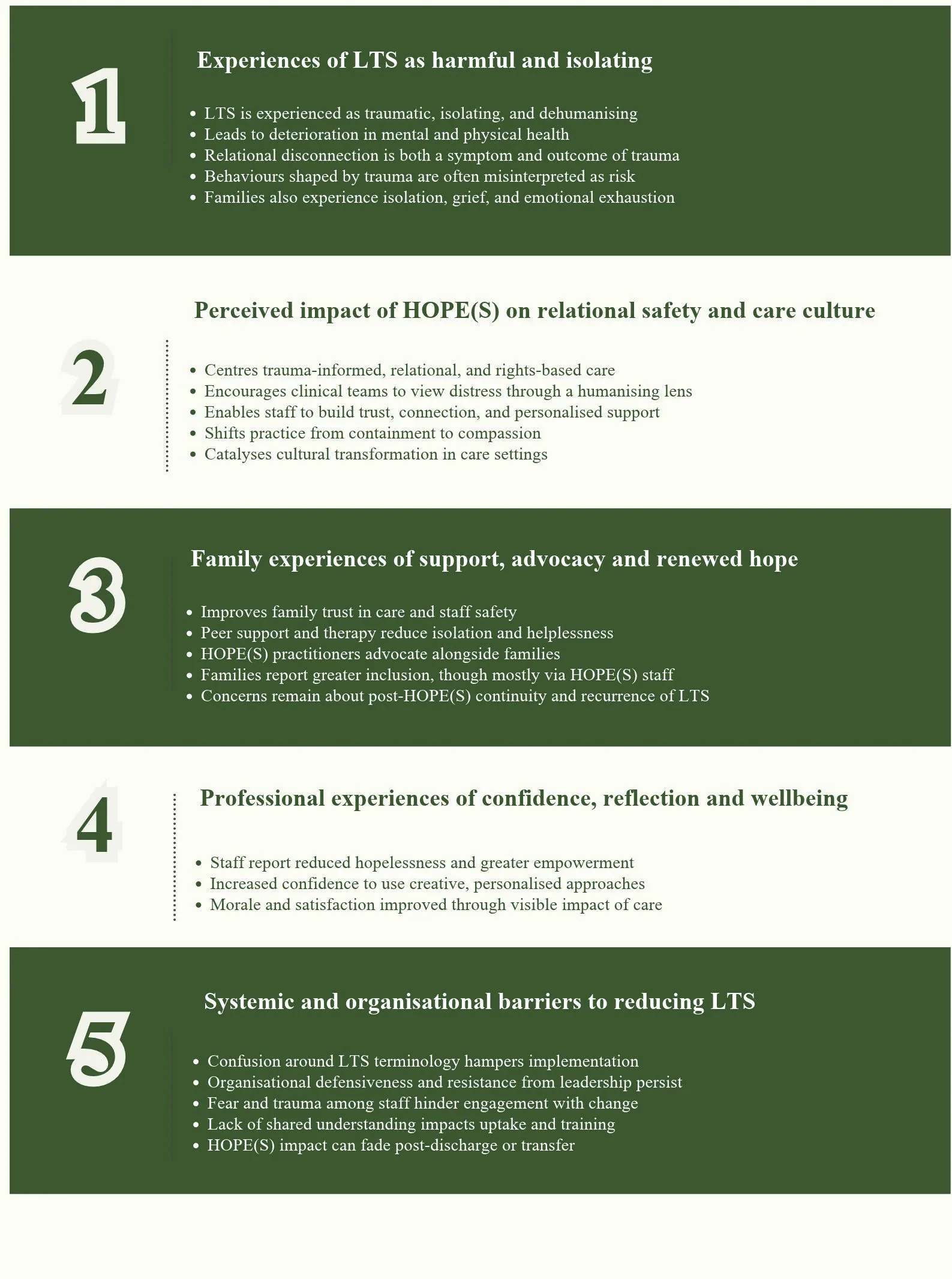
Qualitative themes. Five interrelated themes identified through reflexive thematic analysis of qualitative data from stakeholders involved in or affected by HOPE(S): experiences of LTS as harmful and isolating; perceived impact of HOPE(S) on relational safety and care culture; family experiences of support, advocacy and renewed hope; professional experiences of confidence, reflection and well-being and systemic and organisational barriers to reducing LTS. LTS, long-term segregation.

### Experiences of LTS as harmful and isolating

Across stakeholder groups, LTS was described as a deeply traumatic, dehumanising and isolating experience. Individuals with lived experience reported a deterioration in mental and physical well-being, loss of autonomy and profound social disconnection. Participants described limited meaningful human contact, lack of stimulation and environments experienced as punitive rather than therapeutic.

I think you’re very much ignored considering you’re in a place where you’re being supervised 24/7, you’re still ignored quite a lot or a lot of the time it’s just negative comments being thrown at you*.* (participant 64, lived experience).

Participants described that, as their mental health deteriorated, restrictions increased and opportunities for engagement with staff reduced. Individuals reported increased fear of human contact, distrust of staff and profound social isolation, while behaviours arising in this context were understood by staff primarily through the lens of risk or diagnosis.

One family member also shared, with consent, an account written by their person in LTS. This described how prolonged isolation had progressively disconnected them from the outside world, affected their ability to communicate and relate to others and contributed to profound loneliness, fear and a sense of being excluded from ordinary human relationships. The account also conveyed a lasting alteration in the individual’s sense of self, with the experience of LTS described as continuing to shape how they understood themselves and the world around them.

Equally, family members describe strong feelings of isolation (from the child/individual in LTS, from the wider family and friends, work colleagues), hopelessness, trauma and emotional exhaustion, extending beyond the individual in LTS to their wider family and social relationships.

It’s exhausting. It’s so upsetting to see your child in this situation and to see them slowly just… I just call it a rotting of the soul. It just kills your soul being in such a place. […] your child is segregated, but so are you. No one… there’s no one in my normal life. There’s no one at work, there’s none of my friends, there’s you know… there’s no one who understands this at all*.* (participant 76, family member)

### Perceived impact of HOPE(S) on relational safety and care culture

Participants described HOPE(S) as shifting practice away from a primary focus on containment toward relational and trauma-informed approaches. Clinical staff reported increased confidence in engaging with individuals in LTS, greater emphasis on understanding distress and more personalised responses to risk; for example, staff reported being less likely to view individuals primarily in terms of danger and more in terms of distress.

It started with the shift, instead of being fearful of the gentleman, how to be caring, how to look after him, how to enable him*.* (participant 53, professional: clinical staff)

Commissioners and practitioners described HOPE(S) as influencing wider team culture, including language used to describe individuals and expectations about what change was possible.

The HOPE(S) model transformed the way clinical teams thought about individuals*.* (participant 45, professional: commissioner).

### Family experiences of support, advocacy and renewed hope

Family members described changes in their own well-being alongside perceived improvements in the quality of life of individuals in LTS. Families reported feeling heard and supported for the first time. Peer-support and trauma-informed therapy helped process grief, isolation and helplessness. There was a strong feeling of healing through validation. Those engaged with the peer support group felt like ‘comrades in arms’, as their entire experience was perceived as a war, a constant fight. HOPE(S) practitioners advocated alongside families, who previously felt alone in a hostile system.

His staff are amazing, I can relax that he’s not going to get hurt, that’s a big thing for me*.* (participant 15, family member).

Hope is what it means to us. So, the fact that they go in, they work there, it’s all about least restrictive. It’s about working with the person, the individual. They’re amazing*.* (participant 17, family member)

However, some families noted that their involvement with clinical teams remained indirect and expressed concerns about the recurrence of segregation once HOPE(S) ended and the lack of long-term relational continuity.

I don’t really know what goes on beyond the scenes (participant 76, family member)

I don't think that should be the only time that we can use HOPE(S)…I genuinely feel that if somebody has been segregated and then the team have supported them, it’s great when they get to a position where they don't necessarily need HOPE(S) there all the time, but I think it should still be accessible. It’s a bit like it could prevent that relapse and going back in*.* (participant 17, family member)

### Professional experiences of confidence, reflection and well-being

Staff described feeling less ‘stuck’ and more empowered. Participants reported increased opportunities for reflection, creativity and relational engagement with individuals in LTS, alongside improved morale and job satisfaction.

…she [the HOPE(S) practitioner] gave us the confidence and the knowledge to try different things. (participant 34, professional: clinical staff).

Several staff linked these changes to renewed alignment with professional values such as compassion, empathy and ethical care.

### Systemic and organisational barriers to reducing LTS

Despite perceived benefits, participants identified persistent barriers to reducing LTS, including inconsistent definitions, organisational defensiveness, risk-averse cultures and limited leadership engagement. Participants described fear, trauma and risk-averse organisational cultures as factors that limited staff engagement.

…I think there’s just so many different terms that they’re using now. I wonder if that’s some of the confusion with staff as well when it’s getting called different things in different places*.* (participant 73, professional: commissioner).

HOPE(S) practitioners highlighted challenges in sustaining change within broader systems not fully aligned with relational and rights-based approaches.

…we need to keep an open mind that that might not be because they have negative attitudes. It might just be that they’re anxious and paralysed. So, it’s around nurturing, making sure that the system within the service is a nurturing one. Open I think they call it, open and flexible safety culture and just a learning culture […] How do you change that much wider system? I’m one person going in*.* (participant 3, HOPE(S) practitioner)

Some participants felt that poor understanding of the HOPE(S) model hindered training uptake and internal communication in some settings. Families also reported that relational safety created through HOPE(S) was lost following transfers or discharge.

## Discussion

This mixed-methods evaluation found that implementation of a rights-based, relational intervention was associated with reductions in LTS and other restrictive practices, alongside improvements in some individual and staff outcomes and perceived benefits for families. Quantitative findings showed reductions in physical restraint, chemical restraint and seclusion, increased access to fresh air and meaningful activity and improvements in quality of life among the small subgroup with complete paired Mini-MANS-LD data, with a substantial proportion of individuals moving out of LTS during the evaluation period. Qualitative findings contextualised these outcomes, describing LTS as a profoundly isolating and harmful experience, and HOPE(S) as supporting relational safety, shifts in care culture and renewed hope for families and staff, while also highlighting persistent systemic barriers to sustained change. These findings align with national concerns regarding the harms of prolonged segregation and the need for alternatives that move beyond containment-focused responses.^10 11^

This study has several strengths. It is, to our knowledge, the first to examine LTS in depth within a mixed-methods evaluation of a nationally implemented intervention. Co-production and meaningful patient and public involvement strengthened the relevance and credibility of the findings, consistent with calls for greater lived experience leadership in mental health research and service reform.^4^ The evaluation was embedded within real-world service delivery, enhancing ecological validity.

However, limitations must be acknowledged. The pre-post, single-group design without a comparator limits causal inference, and missing data reduced sample sizes for some quantitative analyses. Variability in routine and quantitative outcome measures data collection and follow-up timing reflects the realities of system-level implementation but constrains standardisation. Ethical considerations precluded experimental designs or recruitment of individuals currently detained in inpatient settings,^31^ shaping both the study design and participant sample. Outcome availability varied substantially, with particularly small complete-pair samples for some analyses, including Mini-MANS-LD quality of life (n=9), and high loss to follow-up for staff ProQOL-5 outcomes. These findings should therefore be interpreted cautiously. Although HOPE(S) was implemented nationally, the evaluation sample was service-based and should not be interpreted as nationally representative. Participant-level geographic information was not collected or reported because the small and clinically distinctive population in LTS created a risk of identification. The geographic representativeness of the evaluation sample therefore cannot be determined.

This evaluation confirms and expands on previous literature documenting the harms of coercion and segregation.^32^ The findings extend prior research that has largely focused on seclusion and restraint by addressing LTS as a distinct, entrenched and poorly understood practice. They are also consistent with our subsequently published qualitative analysis of this dataset, which described dehumanisation, psychological and relational harm, loss of hope and human-rights concerns associated with LTS.^6^

These findings offer a stark confirmation of what people with lived experience, families and advocacy organisations have long described: LTS can be profoundly harmful to individuals and there is an urgent need to reduce it.^10 11 14^ Taken together with the wider literature, the findings add to evidence that prolonged segregation may be experienced as non-therapeutic and can compound trauma, exclusion and barriers to recovery.

There is an urgent imperative to prevent and reduce LTS, with accountability for ensuring quality of life when segregation cannot be immediately avoided. The psychological and emotional toll of LTS also extends beyond the individual. Family members in this study described persistent trauma, helplessness and isolation. Families reported feeling excluded from care planning, disbelieved by professionals and stigmatised by association. They felt HOPE(S) practitioners helped them be more visible, advocating alongside them, rather than being questioned or marginalised. This relief from advocacy fatigue and systemic mistrust aligns with longstanding concerns about how families of individuals in restrictive settings are often excluded or blamed.^9 11^ The HOPE(S) model’s relational and rights-based approach provided an alternative narrative; one where families were seen as partners, not problems. However, family contact with clinical teams was often indirect, and concerns were raised about continuity once HOPE(S) involvement ended. This underscores the importance of embedding sustainable relational safety into core clinical systems, rather than locating it solely within an external intervention. Among the eight family members with repeated SCORE-15 data, none met the specified criteria for clinically significant or reliable improvement in family functioning. Given the very small sample, this finding should be interpreted cautiously and does not establish an absence of benefit to family well-being; qualitative accounts nevertheless indicated that some family members experienced validation, support and renewed hope.

The findings showing improvements in staff compassion satisfaction, alongside reductions in burnout and secondary traumatic stress suggest potential benefits for staff well-being and organisational culture. Staff perspectives also appeared to shift from ‘risk aversion’ and containment towards greater confidence in relational, trauma-informed practice and renewed engagement with professional values such as compassion, empathy and connection, often overshadowed by fear and institutional inertia. This aligns with wider literature indicating that restrictive practices may adversely affect staff morale and contribute to moral injury.^33^ Although improved staff well-being may have implications for workforce sustainability, the present evaluation did not assess outcomes such as staff turnover, sickness absence or absenteeism.

The findings suggest that HOPE(S) may support individuals in LTS to regain opportunities, relationships, choice and trust that restrictive environments/cultures can suppress. We argue that these findings can be interpreted using the Capabilities Approach as an analytic lens,^18 19^ which shifts attention from symptom reduction or risk containment toward individuals’ substantive freedoms to live lives they have reason to value. From this perspective, reductions in restrictive practices and increases in meaningful activity and access to fresh air can be understood as expanded opportunities for agency, dignity and relational connection, rather than solely procedural improvements. Qualitative accounts suggest that relational safety, characterised by trust, understanding and consistency, was central to perceived change, echoing trauma-informed care literature that highlights the role of relationships in reducing coercion.^2 4^

For clinicians, the findings underscore the importance of reflective practice, relational leadership and workforce support. For policymakers and commissioners, they highlight the need to invest in system-level approaches that address organisational culture and conditions, rather than relying solely on regulatory oversight to reduce coercive practices.^11^ This interpretation is also consistent with the IC(E)TR External Oversight Group Report,^13^ which identified continued delayed discharge, weak accountability and difficulties translating independent review recommendations into sustained change in people’s lives.

Our proposed ‘Capabilities- and human rights-based framework to ending LTS’ (figure 3) aligns with the UN CRPD in promoting autonomy, choice and participation.^17^ We also argue that, in line with the capabilities approach,^34 35^ quantitative outcomes should be considered alongside outcomes that reflect what people themselves value in life and from service provision. HOPE(S) combines practitioner coaching, family support and co-produced outcomes within a wider system-level approach.

**Figure 3:**
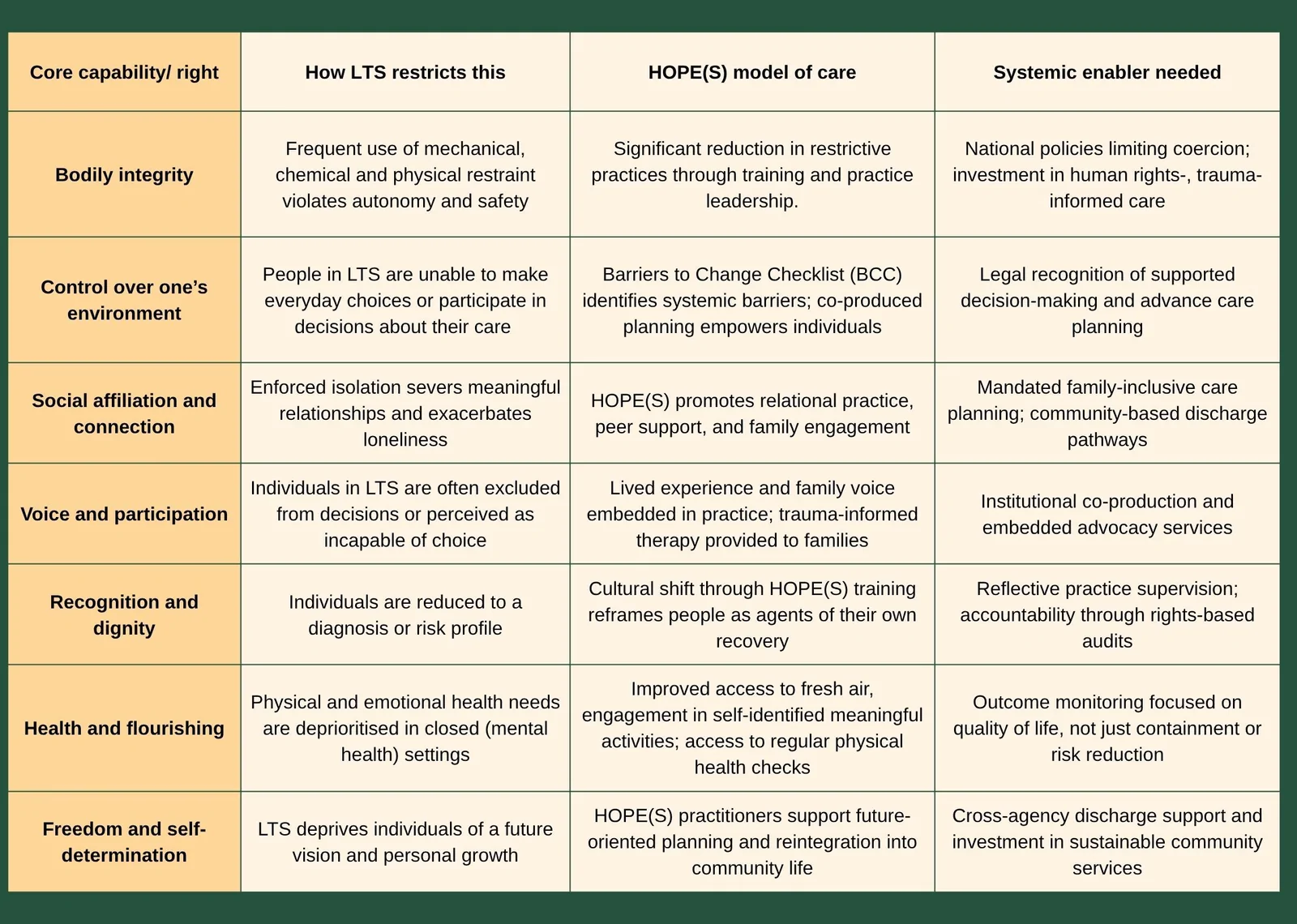
A Capabilities-, Human Rights-Focused Framework to Ending LTS. Conceptual framework informed by the study findings and the Capabilities Approach, illustrating how relational safety, autonomy, choice, participation and opportunities for meaningful activity and connection can support rights-based approaches to reducing long-term segregation. LTS, long-term segregation.

Several unanswered questions remain. Further research is needed to examine the sustainability of changes following the end of HOPE(S) involvement, including longer-term outcomes for individuals, families and staff. Comparative and longitudinal studies could strengthen understanding of which components of relational, rights-based interventions are associated with change and how they can be embedded within routine practice. Research is also required to explore how similar approaches might be adapted across different settings and how lived experience leadership can be meaningfully integrated into system reform. Addressing these questions will be essential to reducing reliance on LTS and advancing humane, effective mental healthcare.

## Conclusions

This study suggests that service reform rooted in rights and capabilities may support dignity, reduce reliance on restrictive practices and promote more relational approaches to care. Our findings are consistent with the latest WHO^16^ guidelines that call for eliminating coercion, investing in community-based care and centring lived experience in service reform. They also align with CRPD mandates for autonomy, legal capacity and non-discrimination. The findings contribute empirical support to critiques that prolonged segregation may reflect systemic and organisational factors as well as individual clinical need.

As countries seek to align with WHO and CRPD guidance, this evaluation indicates that reductions in LTS and other restrictive practices can occur alongside rights-based, individualised approaches supporting relational safety, dignity and recovery.

HOPE(S) provides one example of how training, structured assessment of barriers to change, practitioner support and family involvement can be combined within a system-level intervention. Further evaluation is required to establish the sustainability, transferability and effectiveness of this approach across other services and settings, where restrictive practices are still routinely used.^36^ Additionally, embedding the Capabilities Approach within clinical evaluation frameworks may help redefine ‘success’ in a way that foregrounds dignity and autonomy.

While designed for England, its core components - trauma-informed relational practice, practitioner coaching and family support - may have relevance to other contexts in which restrictive practices are used.

Mental health systems should prioritise the development of open, relationally safe cultures where coercion is not seen as inevitable. Commissioning structures must also enable sustained investment in workforce development, practice leadership and family engagement. Crucially, individuals with lived experience and their families must be central to the design and oversight of such interventions.

The findings reinforce the importance of locating change not solely within the individual but also within the system, relationships and environments surrounding them. What happens next will determine whether we continue a legacy of coercion or commit to systemic justice.

“We need the lifeboats. We need them, and it will… you can’t judge that by metrics. You can’t… which is why I think your work is so important, to hear the individual stories because it’s not something you can put on a graph. It’s not about how many people they’ve managed to get out within six months or… that’s just not the way this works. These are human beings who have been so damaged by an inhumane system that we need to have some… we need to have the lifeboats, otherwise we’re all drowning.” (participant 76, family member)

## Supplementary material

10.1136/bmjopen-2025-115706 online supplemental file 1

## Data Availability

Data may be obtained from a third party and are not publicly available.
