# Supplementary material for: Mixed-methods evaluation of a rights-based intervention to reduce long-term segregation in mental health services: the HOPE(S) National Programme in England
Source: BMJ Open. 2026 Oct 15;16(10):e115706. doi: 10.1136/bmjopen-2025-115706 (PMC13691852; doi:10.1136/bmjopen-2025-115706)
Supplement: Supplementary file 1 [file bmjopen-2025-115706.reviewer_comments.pdf]

PEER REVIEW HISTORY

BMJ Open publishes all reviews undertaken for accepted manuscripts. Reviewers are asked to complete a checklist review form and are provided with free text boxes to elaborate on their assessment. These free text comments are reproduced below.

ARTICLE DETAILS

Title (Provisional)

A Mixed-Methods Evaluation of a Rights-Based Intervention to Reduce Long-Term Segregation in Mental Health Services: The HOPE(S) National Programme in England

Authors

Haines-Delmont, Alina; Fradley, Kathryn; Rajan, Dineesha Georgeena; Cavanagh, Dawn

---

VERSION 1 - REVIEW

**Reviewer**	**1**
**Name**	**Fares, Souha**
**Affiliation**	**American University of Beirut, Hariri School of Nursing**
**Date**	**06-Apr-2026**
**COI**	**None**

---

1. The authors describe this as “the first national evaluation,” which may be overstated. Given the sample size (122 quantitative participants and 73 qualitative participants), it is unclear whether the study can be considered national in scope. Please clarify whether this refers to the evaluation of a nationally implemented intervention rather than a nationally representative evaluation. Additionally, the manuscript should specify the geographic distribution of participants and discuss whether the sample is representative of the broader population across the country.

2. There is some confusion in how the qualitative sample is reported. The abstract mentions 73 participants. In the Methods, you report 49 interviews, 8 focus groups, and 2 written interviews with 73 stakeholders. Please clarify whether the 73 refers to unique participants, and how they are distributed across interviews and focus groups (e.g., number per focus group). This will improve clarity and consistency.

3. The manuscript describes this as the first national evaluation, yet the study is presented as exploratory with no pre-specified hypotheses. Please clarify this positioning. If the study is exploratory, this should be stated more consistently and the claim of a “national evaluation”

should be justified (e.g., whether it refers to a nationally implemented intervention rather than a nationally representative study).

4. There is no information on the reliability and validity of the instruments used. Please report whether these measures are validated and provide references where appropriate. In addition, no reliability results (e.g., internal consistency) are reported for the current sample, which should be included.

5. Figure 3 is missing on page 16.

**Reviewer**	**2**
**Name**	**Markham, Sarah**
**Affiliation**	**King's College, London, Biostatistics**
**Date**	**18-Apr-2026**
**COI**	**None**

Clinically relevant paper

**Reviewer**	**3**
**Name**	**Long, Janet**
**Affiliation**	**Australian Institute of Health Innovation, Australian Institute of Health Innovation**
**Date**	**05-Aug-2026**
**COI**	**None**

This well executed mixed methods study examined the impact of long term segregation practices on mental health in-patients, family members and staff, and evaluated pre- and post implementation of the HOPE(S) program designed to reduce restrictive practices. Data collected is comprehensive and the program clearly had an impact on both patients + families and the staff. The use of the Capability Framework also added value to the findings.

The authors are to be commended for including people with lived experience at every part of the study development. Choosing outcomes that matter to the focal group and being responsive to their recommendations for recruitment and participation are often lacking in this field. Clearly reported too.

This may have been obvious to the authors but I was not sure what in-patient settings you were talking about at first. Can you clearly describe acute mental health in-patient services for an international audience who may only be familiar with acute medical in-patient services. Also, a more meaningful description of the setting in which a person restrained under LTS is kept would be helpful (probably a range of settings across different facilities).

I did not at first realise that so much detail was relegated to Supplementary files. It would be helpful to spell out what exactly is covered in the Supplementary files. For example, the detail of the recruitment and conduct of interviews and focus groups is comprehensively covered there but in the text it looked to me like you had not reported it. Can you have a look at this and make sure that readers know where you have summarised things and where to find the details.

Did some of the same participants take part in the qualitative and quantitative components or were they different pools of people?

Results:

Table 1: It would assist with the reading of the table if significant results (e.g., in the p-value column) were in bold.

Page 9 line 32ff "following HOPE(S) intervention, there were significant reductions in the use of physical restraint, chemical restraint and seclusion". The caption on Table 1 implies otherwise. Can you please clarify?

On page 11, quote from line 44 – There is no clear attribution to a participant (e.g., a number). Is this account of LTS referring to the same participant as the next quote? I note the distinctive use of the word "rot" in both. While these may be the most vivid quotes, they are n=1 and are both the viewpoint / filtered by family member 76. Would be good to find a primary source to replace one of these – a different participant's words for their experience.

Line 40 on p9. Is the word Respond meant to be there?

Figure 1 – can you please add a legend for the non-standard abbreviations such as CRPD.

VERSION 1 - AUTHOR RESPONSE

	**Reviewer: 1**	
	The authors describe this as "the first national evaluation," which may be overstated. Given the sample size (122 quantitative participants and 73 qualitative participants), it is unclear whether the study can be considered national in scope. Please clarify whether this refers to the evaluation of a nationally implemented intervention rather than a nationally representative evaluation. Additionally, the manuscript should specify the geographic distribution of participants and discuss whether the sample is representative of the broader population across the country.	We agree that the previous wording could imply national representativeness. We have therefore revised the manuscript throughout to distinguish between evaluation of a nationally implemented programme and a nationally representative evaluation. HOPE(S) was implemented across England, but the evaluation sample was service-based and should not be assumed to be nationally representative. Geographic information at participant level was not collected/reported because, given the very small and clinically distinctive population in LTS in England, this could increase the risk of identification. We have also added this limitation to the Discussion.

	There is some confusion in how the qualitative sample is reported. The abstract mentions 73 participants. In the Methods, you report 49 interviews, 8 focus groups, and 2 written interviews with 73 stakeholders. Please clarify whether the 73 refers to unique participants, and how they are distributed across interviews and focus groups (e.g., number per focus group). This will improve clarity and consistency.	Thank you. We have clarified that the qualitative sample comprised 73 unique participants. The Methods now report that data were collected through 50 interviews, nine focus groups and two written responses, and the Supplementary Material provides the participant-group breakdown and composition of the focus groups (SM 4.3).
	The manuscript describes this as the first national evaluation, yet the study is presented as exploratory with no pre-specified hypotheses. Please clarify this positioning. If the study is exploratory, this should be stated more consistently and the claim of a "national evaluation" should be justified (e.g., whether it refers to a nationally implemented intervention rather than a nationally representative study).	We have revised the positioning of the study throughout the manuscript. The study is now described as an evaluation of a nationally implemented intervention rather than a nationally representative "national evaluation", and its exploratory, real-world pre-post design is stated more consistently. Claims regarding national scope have been revised accordingly.
	There is no information on the reliability and validity of the instruments used. Please report whether these measures are validated and provide references where appropriate. In addition, no reliability results (e.g., internal consistency) are reported for the current sample, which should be included	Thank you. We have expanded the Measures section to describe the available evidence regarding the reliability and validity of the Mini-MANS-LD, ProQOL-5 and SCORE-15 and have added the relevant validation references. We have also clarified limitations in the applicability and psychometric evidence for these measures in the populations studied. Where current-sample reliability estimates were not available or were not appropriate because of the small analysed samples, we have not generated retrospective estimates and have made this limitation explicit.
	Figure 3 is missing on page 16.	Thank you for identifying this omission. We have checked the revised submission files to ensure that Figure 3 is included, correctly numbered and visible in the manuscript/figure file.
	**Reviewer 2**	
	Clinically relevant paper	Thank you for this encouraging assessment of the clinical relevance of the work.
	**Reviewer 3**	
	This well executed mixed methods study examined the impact of long term segregation practices on mental health in-patients, family members and staff, and evaluated pre- and post implementation of the HOPE(S) program designed to reduce restrictive practices. Data collected is	Thank you for these helpful comments and for recognising the contribution of people with lived experience to the development, conduct and interpretation of the study.

	comprehensive and the program clearly had an impact on both patients + families and the staff. The use of the Capability Framework also added value to the findings. The authors are to be commended for including people with lived experience at every part of the study development. Choosing outcomes that matter to the focal group and being responsive to their recommendations for recruitment and participation are often lacking in this field. Clearly reported too.	
	This may have been obvious to the authors but I was not sure what in-patient settings you were talking about at first. Can you clearly describe acute mental health in-patient services for an international audience who may only be familiar with acute medical in-patient services. Also, a more meaningful description of the setting in which a person restrained under LTS is kept would be helpful (probably a range of settings across different facilities).	Thank you. We have clarified the nature of the inpatient settings for an international readership. In the Methods, we now explain that HOPE(S) was delivered across specialist NHS-commissioned psychiatric inpatient services in England and distinguish these from acute medical inpatient care. We have also expanded the Background description of LTS to explain that its physical arrangements vary across services and may involve separation from other patients within a ward or unit, with very limited peer contact, reduced social interaction and restricted access to ordinary activities.
	I did not at first realise that so much detail was relegated to Supplementary files. It would be helpful to spell out what exactly is covered in the Supplementary files. For example, the detail of the recruitment and conduct of interviews and focus groups is comprehensively covered there but in the text it looked to me like you had not reported it. Can you have a look at this and make sure that readers know where you have summarised things and where to find the details.	We agree that the previous manuscript did not sufficiently signpost the detailed material provided in the Supplementary Material. We have therefore added clearer cross-references throughout the Methods, including explicit signposting to supplementary information on recruitment, qualitative data collection and conduct, participant composition, quantitative data availability and missingness, and the interview/focus-group topic guides.
	Did some of the same participants take part in the qualitative and quantitative components or were they different pools of people?	Thank you. The quantitative and qualitative components were not designed as formally linked cohorts. Some overlap may have occurred, particularly among clinical staff who completed the ProQOL and also participated in a qualitative interview or focus group; however, participant identifiers were not collected in a way that permitted linkage across the two datasets in order to protect anonymity. We have added this clarification to the Methods (Design and Participants section).

	Results: Table 1: It would assist with the reading of the table if significant results (e.g., in the p-value column) were in bold.	Thank you. Statistically significant p-values are now shown in bold in Table 1 to improve readability.
	Results: Page 9 line 32ff “following HOPE(S) intervention, there were significant reductions in the use of physical restraint, chemical restraint and seclusion”. The caption on Table 1 implies otherwise. Can you please clarify?	Thank you for highlighting the potential inconsistency. We have rechecked the restrictive-practice analyses against the statistical output and revised Table 1, its footnotes and the accompanying Results text so that the findings are reported consistently. Physical restraint and seclusion showed significant reductions in the paired analyses; chemical restraint is reported using the Wilcoxon signed-rank analysis because of the non-normal distribution, while mechanical restraint did not show a statistically significant change.
	Results: On page 11, quote from line 44 – There is no clear attribution to a participant (e.g., a number). Is this account of LTS referring to the same participant as the next quote? I note the distinctive use of the word “rot” in both. While these may be the most vivid quotes, they are n=1 and are both the viewpoint / filtered by family member 76. Would be good to find a primary source to replace one of these – a different participant’s words for their experience.	We agree that the original presentation could imply that this quotation was obtained directly from a study participant and could also appear repetitive alongside the following family-member quotation. We have therefore removed the verbatim quotation and replaced it with a prose summary that explicitly states that the account was written by the individual in LTS and shared by a family member with consent. This preserves the relevant experiential material while distinguishing it clearly from directly collected participant quotations.
	Results: Line 40 on p9. Is the word Respond meant to be there?	Thank you for identifying this lack of clarity. We have revised the text to explain that Respond is a specialist charity that provided trauma-informed therapeutic support to family members as part of the wider HOPE(S) programme, and have introduced Respond more clearly when it first appears in the manuscript.
	Results: Figure 1 – can you please add a legend for the non-standard abbreviations such as CRPD.	Thank you. We have added a legend to Figure 1 defining the non-standard abbreviations used in the figure, including CRPD (Convention on the Rights of Persons with Disabilities), BCC (Barriers to Change Checklist), KPI (key performance indicator), LTS (long-term segregation), NHS (National Health Service) and QoL (quality of life).
