# Supplementary material for: Mixed-methods evaluation of a rights-based intervention to reduce long-term segregation in mental health services: the HOPE(S) National Programme in England
Source: BMJ Open. 2026 Oct 15;16(10):e115706. doi: 10.1136/bmjopen-2025-115706 (PMC13691852; doi:10.1136/bmjopen-2025-115706)
Supplement: Supplementary file 2 [file bmjopen-16-10-s001.docx]

# **Supplementary** **material (SM)**

## **SM1: Detailed description of National HOPE(S) Programme to End Long-Term Segregation (LTS) for Children and Young People, Autistic Adults and Adults with a Learning (Intellectual) Disability in Mental Health Inpatient Settings**

The National HOPE(S) Programme was built on a clinical model of care known as the HOPE(S) model (1). This was originally developed by a large mental health and physical health organisation in the North-West of England, to reduce LTS for adults in high secure settings. The HOPE(S) model is based on a philosophy of person centred, human rights-based care, which includes being unconditionally and relentlessly positive. It allows clinical teams and the person in segregation to understand the significant barriers and systemic issues maintaining their circumstance. The HOPE(S) model aims to end or reduce time spent in LTS, improve quality of life and personal experiences, support physical health outcomes, increase family involvement, reduce other restrictive practices and length of stay, support appropriate forward planning and discharge and also change clinical practice to a more human rights-based culture of care.

The HOPE(S) model implements three key components to reduce or end LTS: (1) the HOPE(S) training, (2) the Barriers to Change Checklist (BCC) and (3) intervention by the HOPE(S) practitioners for the person and their staff team. HOPE(S) training includes 2-day clinical teams training and HOPE(S) awareness training. The aim of the training is to raise awareness of the negative impact of LTS through evidence-based literature, case studies and reflective practice, as well as provide staff with an understanding as to what the HOPE(S) model is and what it can offer. The training is facilitated by HOPE(S) practitioners. They support clinical teams to implement the model through practice leadership, advocating for least restrictive practices and encouraging staff to create opportunities to build meaningful relationships, promote meaningful activities and fresh air for those in LTS. The Barriers to Change Checklist (BCC) is a tool used by practitioners, clinical teams and the person in LTS to identify factors that interfere with someone moving out of LTS. The BCC offers a framework for teams to understand dynamic risk factors associated with LTS and help them develop an implementation plan that enables a targeted positive approach for the person in LTS. Four main domains have been identified from research, a thematic analysis of clinical practice and the literature which contribute to and/or act as barriers to moving out of LTS in services and these are assessed and specifically targeted by the BCC.

In 2021 the National HOPE(S) programme was funded to implement the model across England for autistic people, and/or people with a learning disability, and all children and young people in LTS, regardless of diagnosis. Service delivery started in May 2022 due to funding arrangements and recruitment delays (mainly due to Covid-19 organisational staffing issues) and was ceased on 31st March 2025. In collaboration with NHS England (NHSE), the programme developed a prioritisation criterion for HOPE(S) intervention to ensure those people in LTS experiencing greater levels of restriction were prioritised for support. NHSE regional leads in collaboration with HOPE(S) practitioners from the region met regularly to review referrals and agree prioritisation. The prioritisation criteria consisted of the following key areas; are there safety concerns, does the person have access to fresh air and exercise in an outside space, is the person at risk of escalating up the secure pathway and is the person’s human rights being infringed upon or breached. Where NHSE regional leads had safety concerns, people had lack of access to fresh air and exercise in an outside space, the people were at risk of escalating up the secure pathway and their human rights infringed upon or breached, these people were prioritised for HOPE(S) support.

Since its first referral in May 2022, HOPE(S) has been a dynamic and evolving programme based on the feedback from those engaged with the programme and the ongoing research evaluation. From the onset, the demand to provide HOPE(S) for people in LTS was greater than the capacity available within the programme. The programme thus developed an additional level of intervention in the form of consultancy support to enable those individuals and their clinical teams to receive support from the programme. Two levels of support were offered (supplementary table below) and throughout delivering the programme many people receiving support have moved between intensive support and consultancy support depending on clinical need.

## **SM2: Supplementary table clarifying the two levels of support offered by HOPE(S) according to clinical need**

|  | Intensive support | Consultancy Support |
| --- | --- | --- |
| HOPE(S) practitioner engagement | • Engage with person and assess the salient issues relevant to long-term segregation  • Connect with the family / carers, clinical and ward team, advocacy, and wider stakeholders | • Engage with person and assess the salient issues relevant to long-term segregation  • Connect with the family / carers, clinical and ward team, advocacy, and wider stakeholders |
| Completion of Barriers to Change Checklist (BCC) | • Facilitate the completion of the BCC with the person and clinical team  • Address any urgent human rights / quality of life issues  • Generate 3 SMART key intervention targets | • Facilitate the completion of the BCC with the person and clinical team  • Address any urgent human rights / quality of life issues  • Generate 3 SMART key intervention targets |
| Delivery | • Intensive relational and therapeutic work with the person in LTS  • Train clinical and ward teams in the HOPE(S) model  • Support the teams through coaching, practice leadership, reflective practice, and clinical supervision to achieve the key intervention objectives  • Train wider networks including future community providers | • Train clinical and ward teams in the HOPE(S) model  • Support the teams through reflective practice and clinical supervision to achieve the key intervention objectives |
| Transition | • Monitor progress through experiential narrative and outcome measures  • Develop infrastructure to maintain transition and progress | • Monitor progress through experiential narrative and outcome measures (not including physical health and other quality of life measures) |

## **SM3: Supplementary table detailing the missing data for the paired t-tests***

| **Outcome/Measure** | **Number of responses** | | **Missing data** | |
| --- | --- | --- | --- | --- |
|  | **Pre-HOPE(S)** | **Post-HOPE(S)** | ***n*** | **%** |
| Type of restrictive practice |  |  |  |  |
| *Mechanical* | 59 | 60 | 1 | 1.67 |
| *Physical* | 58 | 60 | 2 | 3.33 |
| *Chemical*** | 57 | 60 | 3 | 5 |
| *Seclusion* | 60 | 59 | 1 | 1.66 |
| Quality of life | 15 | 9 | 6 | 40 |
| Access to fresh air | 60 | 57 | 3 | 5 |
| Meaningful activities | 61 | 56 | 5 | 8.20 |
| Staff's quality of life |  |  |  |  |
| *Compassion satisfaction* | 376 | 64 | 312 | 82.98 |
| *Burnout* | 379 | 68 | 311 | 82.06 |
| *Secondary traumatic stress* | 377 | 68 | 309 | 81.96 |
| Family functioning* | 11 | 8 | 3 | 27.27 |

Note: *Paired t-test was not used for the SCORE-15 (family functioning) as change was analysed descriptively in line with the recommendation by Miller et al., (41).

## **SM4: Supplementary information regarding qualitative data collection and analysis**

### ***SM4.1. Recruitment***

All participants were recruited by the research team using volunteer sampling. Information about participation and how to contact the research team was provided to all people who might have had experience of the National HOPE(S) programme by the practitioners delivering the intervention (i.e., HOPE(S) or Respond practitioners). This included family members, people with lived experience, and professionals (e.g., clinical staff, commissioners, regional managers, regulators). In addition, the research team used other recruitment boosting techniques, for example, at the National HOPE(S) conference in May 2024, having a table to showcase the research and encourage participation.

All eligible participants were given sufficient time to read a participation information sheet and a consent form prior to deciding whether they wanted to participate. Different versions of the participation information sheet were created to tailor the information for each type of participant (family member, person with lived experience of LTS and/or restrictive practices, professionals). Those interested in participating contacted the research team by email.

### ***SM4.2. Consent***

Prior to interviews/focus groups, all participants were required to provide consent either through signing the consent form or by providing verbal consent to the statements presented on the consent form (which were recorded part of their interview/focus group). Data was not analysed nor included in this paper if consent was not given. To ensure anonymity, all participants were given a participant number/code instead of using their names and this was used in this paper when reporting quotes from their data.

### ***SM4.3. Data collection***

Interviews and focus groups were conducted by an independent research team (including authors KF and AH-D). All interviews and focus groups were supervised by the study Chief Investigator (CI), AH-D, a senior researcher. All interviewers had previous experience interviewing and identify as a female based on the authors’ current knowledge. A sample of interviews were supervised by the study CI to ensure consistency in data collection. Participants did not have a previous relationship with the researchers, and participants were made aware of the researchers’ role and the project aims.

An interview guide was used for interviews and focus groups, but this was tailored to the type of participant or circumstances (see SM4.4 below). Participants were provided with a summary of example questions prior to attending the interview and/or the focus group. For example, family members were asked to share their thoughts and feelings about their loved one’s journey into hospital and LTS; views about HOPE(S) and the therapy from Respond (where applicable); benefits and challenges and ideas for improvement. Respond therapists were asked about their role in relation to HOPE(S), specifics with regards to Respond therapy and engaging with families, key benefits, and challenges. HOPE(S) practitioners were asked to share their views and experiences about HOPE(S), anything from rationale for LTS, specifics to do with their role, good practice, challenges, and ideas on how to improve or enable sustainable practice.

Qualitative data were collected between July 2023 and January 2025 through semi-structured interviews and focus groups, using topic guides adapted to participants’ roles and experiences. Data collection occurred in two waves, with the second wave informed by findings from the initial HOPE(S) evaluation. The first wave (July 2023 - January 2024) primarily involved interviews with family members and HOPE(S) practitioners and focused on experiences of HOPE(S) implementation. The second wave (April 2024 - January 2025) expanded recruitment across clinical staff, managers, commissioners, regulators, families and people with lived experience, using interviews and focus groups to explore barriers associated with LTS and its continuation.

In total, 73 individuals contributed qualitative data. Some participants contributed to more than one session; these individuals were counted once in the overall sample. A total of 50 interviews were conducted with HOPE(S) practitioners (n = 10), family members (n = 11), clinical staff (n = 19), other professionals (n = 6), and Respond practitioner (n = 4). Two family members completed written responses due to accessibility needs.

A total of nine focus groups were conducted:

- Four focus groups consisted of only clinical staff (n = 10)
- One focus group consisted of Respond practitioner (n = 3)
- One focus group consisted of commissioners (n = 4)
- One focus group consisted of family member (n = 2)
- One focus group consisted of individuals with lived experience (n = 4)
- One focus group consisted of four clinical staff and a Respond practitioner.

Interviews and focus groups were predominantly conducted online via Microsoft Teams. One focus group involving people with lived experience was conducted face-to-face and audio-recorded. Support workers were present during sessions involving people with lived experience or family members to support wellbeing and provide post-session support. Although different researchers facilitated the interviews and focus groups, including research assistants and associates, the PI provided overall supervision of all qualitative data collection. Researchers also maintained reflexive field notes to document contextual observations and reflections.

### ***SM4.4. Interviews and focus groups schedules and indicative questions***

IRAS ID: 319279

SPONSOR ID: 46401

**STUDY TITLE:** Evaluation of the National HOPE(S) Programme to end long-term segregation (LTS) for all children and young people, autistic people and/or people with a learning disability in inpatient hospital setting.

**SEMI-STRUCTURED INTERVIEW SCHEDULE**

**HOPE(S) Practitioners**

1. **Can you tell me about your experience and current role in relation to the HOPE(S) Programme**

*Prompts: what does the HOPE(S) model mean to you? How does your day-to-day activity contribute to the overall aims of the Hopes model? E.g., helps clinical teams/person in segregation to improve experience/physical health outcomes/reduce length of stay/improve discharge arrangements***,** *aims to achieve person-centred/human rights-based care/positive approach to reducing LTS. Has your impression of HOPE(S) changed throughout – positive and negative?*

2. **Have you found the BCC useful in helping you meet the needs of your priority person?**
3. **Can you tell me a bit about your experience with the HOPES training and whether or not it has made a difference to staff teams?**
4. **What is different about HOPE(S) compared to usual practice?**

*Prompts: examples of HOPES practice, how it was before; how it changed, if it did. Also refer to HOPE(S) practitioner as a new role vs. old job]*

5. **In your view what are the key benefits of implementing the HOPE(S)**

*Prompts: compared to how interventions were previously provided for individuals within LTS (one or two benefits what would they be? Prompts: does it meet the needs of individuals/does it make a difference to them*

6. **Can you tell me about any key challenges you’ve faced?**

*Prompts: working in a different way/MDT approach/relationship with the person in LTS.*

7. **What are you most proud of during your work with a priority person?**

*Prompts: what has been learnt/different approach due to HOPE(S)/relationships. One memorable experience.*

8. **Why do you think that individuals end up in long term segregation?**

*Prompts: a reflective question- tease out thoughts/feelings, Why/how did your person end up in this situation (LTS) in the first place? [understanding of their person’s journey into LTS*

9. **Can you share one thing that you would do differently to improve the implementation/outcomes of HOPE(S)?**

*Prompts: what interventions can they suggest*

10. **In your view how can this be embedded into practice in the future?**

*Prompts: what challenges have they identified and how can their suggestions/ the future model be sustained*

11. **Is there anything else you want to say about HOPE(S)/your experience that hasn’t been covered?**

**SEMI-STRUCTURED INTERVIEW SCHEDULE**

**(RESPOND) Families/Carers**

1. **Can you tell me about your loved one’s journey into hospital and long-term segregation?**

- Prompts*:*
- *How did/does it feel for them?*
- *How did/does it feel for their loved one?*

2. **Who supported you and your family through this journey?**

- Prompts*: main source of support: other family? Friends? Services? None? How do they feel about it?*

3. **What are your views about HOPES? And your model?**

- Prompts: *what does it mean to them? What changed? How is it different from before/’usual practice’?*

4. **How do you/your family feel about receiving therapy from RESPOND?**

- Prompts:
- *How long have they been involved/did they attend all sessions?*
- *One key thing they found beneficial. [example]*
- *Any challenges? [e.g., things with which they have struggled. Did not work that well for them? If they have not engaged that well, ask them why]*

5. **What made you say ‘yes’ to engage with RESPOND?**

- *Prompts: example of 1-2 things they would do to help people engage; also explore the notion of therapy, stigma, trust in services, guilt, fear of re-traumatisation, etc.*

6. **Why do you think people end up in LTS in the first place?**
7. **Any thoughts about the future care and support for your loved one?**
8. **(If time allows): one thing that you would change in practice so people with LD/autism have a better QoL and do not end up institutionalised or in LTS?**
9. **(If time allows): one thing you found useful regarding HOPES/RESPOND –** *[i.e., that really helped them or their loved one while in hospital or their journey from LTS into the community?]*

**SEMI-STRUCTURED INTERVIEW SCHEDULE (written format, n = 2)**

**Families/Carers**

We really want to capture (1) the journey of your loved one in long term segregation, how it was for them, (2) how it was for you, and then (3) how this changed after they became part of the HOPES programme.

Please share your thoughts, feelings and experiences. Tell us how it felt at the time. Give us examples. THANK YOU!

1. How did your loved one end up in long term segregation and how was it for them? Ask them how life was like? How did it make them feel? Please use their own words, if possible
2. How was it for you? how did it make you feel?
3. What changed after they became part of the HOPES programme? How did it make them feel? What about you?
4. What is the one thing you want people to know about HOPE(S)? what was the biggest impact for you?

**SEMI-STRUCTURED INTERVIEW/FOCUS GROUP SCHEDULE**

**Healthcare staff/other professional stakeholders**

**Hands on experience**

- What was your first impression of the HOPES intervention?
- How has your impression/opinion changed over time delivering or receiving the intervention?
- What has been important in keeping you going when facing challenges in the system?
- What are you most proud of in this work to reduce LTS so far?
- What is the most important thing you have learnt from your priority person?

**Views about HOPES**

- Does HOPES meet people’s needs? How? Are there any aspects of LTS care that you feel are not addressed by the HOPES intervention?
- Does HOPES achieve its goals/objectives? [refer to the outcomes in the model, e.g., culture/organisational change, training, etc.] How?
- What are the key benefits? [why] [focus on a key one]
- What are the key challenges and how can they be addressed? [focus on a key challenge]

**Final thoughts (if not covered above)**

- If you could change one thing, what would that be?
- If it were to continue, how can it work?
- Anything else we might have missed or you’d like to add?

**FOLLOW UP INTERVIEWS AND/OR FOCUS GROUPS (ROUND 2)**

**INDICATIVE SCHEDULE FOR PROFESSIONALS (E.G., HOPES AND RESPOND PRACTITIONERS, CLINICAL STAFF, COMMISSIONERS)**

*RULES*

*If this is an interview*

- *allow 5 minutes at the beginning for the person to settle (appreciation for being here, ask them to think why they were interested to speak to you)*
- *restate purpose of the interview and all the rules – remind people that they are invited here because we want to know what they are thinking – describe the agenda for the interview – beginning, questions, end*
- *check if they have read the PIS and signed and sent the consent form*
- *start recording and transcription on Teams*
- *Introductions – name and role*

*If this is a focus group*

- *gallery view – if possible*
- *clock visible on the screen*
- *same as above; the key difference is that I state how we work, e.g., we start we rounds – everybody will speak for 3 minutes, as we are interested in everybody’s thinking, we find that people think better if they are given attention in the room*
- *first clockwise, then counter-clock wise*
- *choice to pass if you don’t want to speak at a particular point during a round*
- *we are interested in quality, rather than quantity – people to be present in the room – so please put your mobile away and switch off all the other running apps on your laptop, including email.*
- *let’s acknowledge what is working well before problem solving what is not working that well.*

**INDICATIVE QUESTIONS PROFESSIONALS**

1. **What is your experience of (using) the HOPES model?**

*[if commissioner: How are you connected to HOPES in your role? Experience regarding the referral process, criteria used, definition/identification of cases of LTS]*

2. **Have you had experience of HOPES training?** *[what was it like? What is their insight into some of the key benefits and limitations?]*
3. **What is your experience of the BCC?** *[what aspects were helpful or less helpful, examples in practice, has it made a difference, in their view?]*
4. **Experience of the practice leadership element of HOPE(S)?** *[examples in practice, has it made a difference? how important a part of the model is it?]*
5. **How is HOPES different to other interventions or usual practice? Example**
6. **What do you see as the key benefits (that haven’t been mentioned) of implementing HOPES in your service?** *[quality of life for patients, staff, meaningful activities, culture change, reduction in restrictions, using alternative interventions other than LTS]*
7. **What have been the key challenges of implementing HOPES within your service?** *[more generally what are the key challenges or limitations with regards to HOPES?]*
8. **In your view, how might HOPE(S) impact on the culture, values and future practice of your organisation***?* **What about you, staff, your colleagues in general?**
9. **In your view, is LTS a necessary practice?** *[based on their experience, changed as a result of HOPE(S)?, suggestions for other interventions instead?] [also what are the things that contribute to individuals ending up in long term segregation?]*
10. **What is the most useful thing you have learnt from engaging in HOPES?** *[one thing that you will do differently in practice]*
11. **In your view how can this be embedded into practice in the future?** *[what challenges have you identified and how can their suggestions/ the future model be sustained?]*
12. **Is there anything else you want to say about HOPE(S)/your experience that hasn’t been covered?**

*At the end*

- *Thanks*
- *Allow 5 minutes to reflect on the interview – what they take away from the meeting;*
- *what they valued about the meeting*
- *Offer your appreciation (for the interviewee)*
- *Ask then to offer appreciation for the meeting or somebody in the meeting (next to them).*

**INDICATIVE SCHEDULE FOR FAMILY MEMBERS/CARERS**

*RULES*

*At the beginning:*

- *allow 5 minutes at the beginning for the person to settle (appreciation for being here, ask them to think why they were interested to speak to you)*
- *restate purpose of the interview and all the rules – remind people that they are invited here because we want to know what they are thinking - describe the agenda for the interview – beginning, questions, end*
- *check if they have read the PIS and signed and sent the consent form*
- *start recording and transcription on Teams*
- *Introductions – name and role.*

**INDICATIVE QUESTIONS FAMILY MEMBERS/CARERS**

1. **Why did you want to speak to us?**
2. **Tell me about your experience of caring for somebody in LTS – during LTS?**
3. **What about them? Do you know how they felt? What they were like? What they missed when they were in LTS? What you missed?**
4. **Who supported you and your family through this journey?**
5. **What are your views about the HOPES programme?** *[one thing you found useful regarding HOPES, e.g., that really helped them or their loved one while in hospital or their journey from LTS into the community]*
6. **Do you know how it felt for them once they came out of LTS? How did it feel for you?**
7. **Do you think such practices are necessary? Why?**
8. **What can be done to prevent this from happening? one thing that you would change in practice so people with LD/autism have a better QoL and do not end up institutionalised or in LTS?**

*At the end*

- *Thanks*
- *Allow 5 minutes to reflect on the interview – what they take away from the meeting;*
- *what they valued about the meeting*
- *Offer your appreciation (for the interviewee)*
- *Ask then to offer appreciation for the meeting or somebody in the meeting (next to them)*

### ***SM4.5. Data analysis***

Recordings were transcribed, then imported into NVivo 14 (2)(Lumivero, 2023) for reading, coding, and analysis. Reflexive thematic analysis (3, 4)(Braun & Clarke, 2021; Braun et al., 2023) was undertaken by members of the research team. KF coded all the transcripts and the other members of the research team (JL, KG, and AH-D) coded samples of the transcripts. Themes were derived from the codes and revised through sense-checking.

Specific techniques were adopted to strengthen the rigor of the results, including (i) peer-debriefing amongst the authors during the initial stages of coding, (ii) member-checking was performed on the emerging findings with individuals with lived experience, family members and professionals who attended the national HOPE(S) conference in May 2024; and, (iii) sense-checking of emerging findings and recommendations with a family member of someone with lived experience of LTS; someone with lived experience of LTS; clinical staff; and members of the HOPE(S) National Group including individuals with lived experience, family members, commissioners and regulators, advocacy groups, independent charity groups, service and intervention providers/staff.

## **SM5. Supplementary information regarding quantitative data collection and analysis**

### ***SM5.1. Data collection***

The research team received data from the HOPE(S) team regarding the cohort of individuals who have received support through HOPE(S). Data collection was overseen by the HOPE(S) clinical business manager and the study CI. Clinical outcome data such as start and end date of long-term segregation, use of restrictive practices and engagement in meaningful activity were (routinely) recorded in contemporaneous clinical notes on electronic patient record systems. These data were shared by provider organisations before being categorised and recorded by HOPE(S) practitioners in an anonymised format. Data collection for the quantitative study started on 3^rd^ May 2022 and ended on 17^th^ March 2025.

### ***SM5.2. Governance***

Provider organisations agreed to the sharing of clinical outcomes data for the purposes of this evaluation covered by the Data Protection Impact Assessment (DPIA) between NHSE and the NHS trust managing the data by signing a Memorandum of Understanding (MoU). The transfer to and use of data by the research team was covered by data protection impact assessments and research ethics approval (IRAS project ID: 319279; REC reference: 23/SS/0044).

### ***SM5.3. Data preparation and analysis***

Data was pseudo-anonymised prior to being sent to the research team, i.e., being stripped of any identifiable information such as: the names of individuals engaged with HOPE(S), family members/carers, clinical staff/professionals; their date of birth (DOB), address, email, or phone number; their job titles; the names of participating organisations, hospitals, or units. The data collected were entered into SPSS software version 29 (5) for all statistical analysis. The dataset underwent a data cleaning process to address data entry errors, recoding, missing values, and outliers.

Descriptive statistics (n, %) were performed to determine how many individuals supported through HOPE(S) have ended LTS, as well as for all the outcome measures. For family functioning (SCORE-15), the cut-off for clinical or reliable improvement in therapy over time was used instead (6)(Miller et al., 2023). Paired t-tests were performed if the sample size was sufficient to determine change in key outcome measures, e.g., individuals’ quality of life, access to fresh air, engagement in meaningful activities and use of restrictive practices (restraint and seclusion) pre- and post- HOPE(S). Assumptions were tested (Shapiro-Wilks test of normality). Participants who did not have data for both pre- and post- HOPE(S) were excluded from paired t-tests analyses.

### ***SM5.4. Data availability statement for quality of life outcome measures***

Professionals completed one of the following measures according to their appropriateness for each individual: the Youth Quality of Life (YQOF, n = 4), Maslow Assessment of Needs Scale (MANS, n = 5), Mini-MANS-LD (n = 15) and the HOPE(S) quality of life tool (HOT) (n = 7)^[1]^. Due to its comparatively larger sample size, only data collected from the Mini-MANS-LD were included in the analysed. Out of the 15 individuals who had a completed Mini-MANS-LD, 9 had both baseline and follow-up data and was therefore, included in the paired t-test analysis.

388 available staff who completed the ProQol to some extent. Out of the 388, 376 completed all the items for the Compassion scale, 377 completed all the items for the Burnout scale, and 379 completed all the items for the Secondary Trauma Scale. At time 2, 64 staff provided complete follow-up data for the compassion scale, while 68 staff provided complete follow-up data for the burnout and Secondary Trauma Scale.

1. HOT - prototype research version. Originally developed by Joanna Brackley and Stephanie Carr

   through the HOPE(S) programme. Copyright/IP ownership under institutional review. Not for routine clinical use.
